# A Review of Scar Scales and Scar Measuring Devices

**Published:** 2010-06-21

**Authors:** Regina Fearmonti, Jennifer Bond, Detlev Erdmann, Howard Levinson

**Affiliations:** ^a^Division of Plastic and Reconstructive Surgery, Department of Surgery; ^b^Department of Pathology, Duke University Medical Center, DUMC 3181, Durham, NC 27710

## Abstract

**Objective:** Pathologic scarring affects millions of people worldwide. Quantitative and qualitative measurement modalities are needed to effectively evaluate and monitor treatments. **Methods:** This article reviews the literature on available tools and existent assessment scales used to subjectively and objectively characterize scar. **Results:** We describe the attributes and deficiencies of each tool and scale and highlight areas where further development is critical. **Conclusion:** An optimal, universal scar scoring system is needed in order to better characterize, understand and treat pathologic scarring.

Scarring affects patients following trauma, burns, and surgical procedures. Several modalities have been devised to quantify scars for the purposes of determining response to treatment and for evaluating outcomes. Scar assessments can be objective or subjective. Objective assessments provide a quantitative measurement of the scar, whereas subjective assessments are observer dependent. Quantitative assessment of scars requires devices to measure their physical attributes. Subjective methods to assess scar provide a qualitative measurement of scar by a patient or clinician. Semiquantitative methods to assess scars have been developed by using scales to make subjective methods more objective. This review article describes and compares the tools and assessment scales used to measure scars and highlights the limitation of existing scales.

## DEVICES TO OBJECTIVELY QUANTIFY SCAR

Scar-measuring devices should be noninvasive, accurate, reproducible, and easy-to-use to facilitate objective data collection and have clinical utility. Existing devices assess parameters such as pliability, firmness, color, perfusion, thickness, and 3-dimensional topography.

### Pliability

Several tools have been applied to assess pliability: the pneumatonometer and cutometer are among the most popular. The pneumatonometer uses pressure to objectively measure skin pliability. It is composed of a sensor, a membrane, and an air-flow system that measures the amount of pressure needed to lock the system.^1^ Application of the pneumatonometer to measure cutaneous compliance (Δ volume/Δ pressure) has yielded statistically significant differences in skin compliance based on body site as well as demonstrated overall less compliance of burn scars in all sites as compared to normal controls.^2^ This suggests potential applicability in objective assessment of scar formation.

The cutometer is a noninvasive suction device that has been applied to the objective and quantitative measurement of skin elasticity.^3^,^4^ It measures the viscoelasticity of the skin by analyzing its vertical deformation in response to negative pressure. It has been used to measure the effects of treatments on burn scars and to assess scar maturation. Draaijers et al^5^ have applied the cutometer to the measurement of scar pliability, finding it to be a reliable tool to measure elasticity and extension characteristics of scar tissue.

### Firmness

The durometer applies a vertically directed indentation load on the scar to measure tissue firmness. Originally described for use in scleroderma,^6^ it has since been applied to the analysis of induration in burn scar assessment, although results have been observed to demonstrate high inter- and intraobserver variability.^7^

### Color

Tools have also been developed to objectively measure scar color. The Chromameter (Minolta, Tokyo, Japan), the DermaSpectrometer (cyberDERM, Inc, Media, PA, USA), the Mexameter (Courage-Khazaka, Cologne, Germany), and the tristimulus colorimeter are among the most widely applicable devices.^8^ These devices use spectrophotometric color analysis to calculate erythema and melanin index. Draaijers et al^9^ compared available devices used to measure and assess scar color: the tristimulus colorimeter, a narrow-band simple reflectance meter, and the chromameter. They concluded that these devices assess vascularity and pigmentation better than subjective rating scales, and while he found the DermaSpectrometer easier to use than the Chromameter, they noted good reliability measurements among both devices.

### Thickness

Ultrasound scanners, such as the tissue ultrasound palpation system (TUPS), have been used to quantify scar thickness. Fong et al^4^ compared ultrasonography to the cutometer in terms of objectively measuring scar maturation, finding both to be more sensitive and specific than analysis by their own clinical rating scales for color and consistency. Results from TUPS analysis have also been compared with those obtained with the Vancouver Scar Scale (VSS), one of the most widely applied scar assessment scales in clinical research (discussed further); TUPS was found to demonstrate a moderate correlation in terms of reliability.^10^ TUPS does have drawbacks, however, in that it requires technical training and experience in image interpretation and is relatively expensive compared to other modalities.

### Perfusion

Laser Doppler perfusion imaging is an established technique for the measurement of burn scar perfusion. It aids in early determination of burn depth and subsequent treatment course.^11^ Through constructing color-coded maps of tissue microperfusion, laser Doppler perfusion imaging offers a noninvasive alternative to burn wound biopsy. Sarov and Stewart^12^ have compared this technique to the newer laser-based method, a speckle decorrelation analysis, which uses photon interference patterns to map blood flow to tissues. The authors have demonstrated a statistically significant correlation between the 2 methods in mapping the same relative changes in tissue blood flow; statistically significant correlations were also noted between both methods and various observed clinical parameters (scar pigmentation, vascularity, pliability, and scar height).

### Three-dimensional topography

Three-dimensional systems are attractive for their ability to capture scar surface characteristics with high definition and reproducibility. Roques et al^13^ used the 3-dimensional optical profiling system (Primos imaging) to generate a high-resolution topographic representation of the scar, finding it to be an effective tool to characterize scar. Taylor et al^14^ applied a noncontact 3-dimensional digitizer in their study of keloids to measure scar volume and response to treatment. Scanned scar volume was comparable to physical assessment as measured by the Visual Analog Scale (discussed further) with scar ranking. They noted a statistically significant correlation between measured keloid volume and scar score. While these more advanced imaging methods hold great promise in objectively analyzing pathologic scars, it has been argued that their added expense makes them more applicable to research rather than clinical assessment and treatment monitoring.^15^

While scar assessment instruments have demonstrated accuracy and reliability in comparative studies, there is still a lack of consensus regarding the most appropriate and applicable evaluation instrument. Refinement of scar assessment methods will facilitate accurate analysis of treatment outcomes and enhance the ability to study scarring.

## SUBJECTIVE SCAR ASSESSMENT SCALES

**Figure 1:**
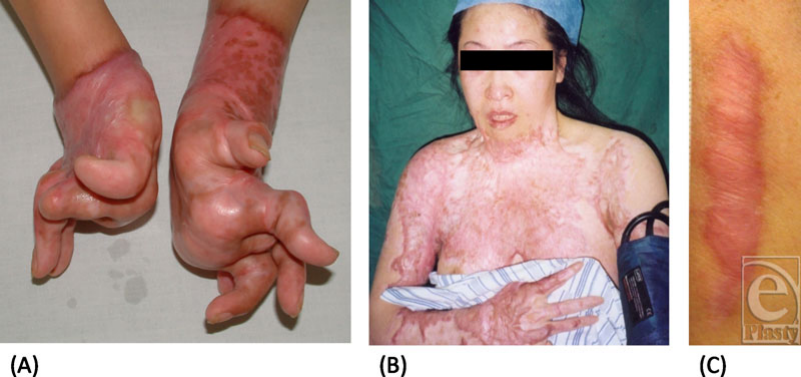
Three different scars are presented to illustrate the wide variety of scarring: (*a*) scarred hands and fingers with contractures, (*b*) large total body surface area scar, and (*c*) a linear scar. Existing scar scales are useful to evaluate linear scarring but not functionally debilitating scarring or large total body surface area scars. (Photos courtesy of Howard Levinson, MD, and Yixin Zhang, MD).

Scar scales have been devised to quantify scar appearance in response to treatment. There are currently at least 5 scar scales that were originally designed to assess subjective parameters in an objective way (Table 1): The Vancouver Scar Scale (VSS), Manchester Scar Scale (MSS), Patient and Observer Scar Assessment Scale (POSAS), Visual Analog Scale (VAS), and Stony Brook Scar Evaluation Scale (SBSES). These observer-dependent scales consider factors such as scar height or thickness, pliability, surface area, texture, pigmentation, and vascularity.^16^ The measurements range across a continuum of values. Thus, the scales are best used to determine change within an individual rather than between individuals. Scar scales are frequently used in research settings and are beneficial to study small, linear scars. Scar scales are only minimally useful for studying large scars and for assessing the functional affects of scarring (Fig 1).

### Vancouver Scar Scale (VSS)

The VSS, first described by Sullivan in 1990, is perhaps the most recognized burn scar assessment method.^16^,^17^ It assesses 4 variables: vascularity, height/thickness, pliability, and pigmentation. Patient perception of his or her respective scars is not factored in to the overall score. Lye et al^18^ compared the pliability score from the VSS to measurements obtained through tonometry, noting moderate correlation in scores. The VSS remains widely applicable to evaluate therapy and as a measure of outcome in burn studies (Table 2).

### Visual Analog Scale (VAS)

The multidimensional VAS is a photograph-based scale derived from evaluating standardized digital photographs in 4 dimensions (pigmentation, vascularity, acceptability, and observer comfort) plus contour. It sums the individual scores to get a single overall score ranging from “excellent” to “poor.” It has demonstrated high observer reliability and internal consistency when compared to expert panel evaluation, but it has shown only moderate reliability when used among lay panels.^19^^-^21

### Patient and Observer Scar Assessment Scale (POSAS)

The POSAS includes subjective symptoms of pain and pruritus and expands on the objective data captured in the VSS.^22^ It consists of 2 numerical numeric scales: The Patient Scar Assessment Scale and the Observer Scar Assessment Scale. It assesses vascularity, pigmentation, thickness, relief, pliability, and surface area, and it incorporates patient assessments of pain, itching, color, stiffness, thickness, and relief. The POSAS is the only scale that considers subjective symptoms of pain and pruritus, but like other scales it also lacks functional measurements as to whether the pain or pruritus interferes with quality of life. Linear regression analysis has demonstrated that the observer's opinion is influenced by vascularization, thickness, pigmentation, and relief, whereas the patient's opinion is primarily influenced by pruritus and scar thickness.^23^ The POSAS has been applied to postsurgical scars and used in the evaluation of linear scars following breast cancer surgery, demonstrating internal consistency and interobserver reliability when compared to the VSS with the added benefit of capturing the patients' ratings^24^ (Table 3).

### Manchester Scar Scale (MSS)

The Manchester Scar Scale, proposed by Beausang et al^25^ in 1998, differs from the POSAS in that it includes an overall VAS that is added to the individual attribute scores. It assesses and rates 7 scar parameters: scar color (perfect, slight, obvious, or gross mismatch to surrounding skin), skin texture (matte or shiny), relationship to surrounding skin (range from flush to keloid), texture (range normal to hard), margins (distinct or indistinct), size (<1 cm, 1-5 cm, >5 cm), and single or multiple.^26^ Scores from the 2 scales are added together to give an overall score for the scar, with higher scores representing clinically worse scars. These data are analyzed in conjunction with details regarding race, ethnic background, history, cause, symptoms, treatments, and responses. Unlike the VSS, the MSS groups together vascularity and pigmentation under the heading of “color mismatch” relative to the surrounding tissue, allowing it to achieve better interrater agreement as compared to the VSS.^27^ It is thus applicable to a wider range of scars and well-suited for postoperative scars. The MSS has not been used in research, however, perhaps because of the wide applicability of the VSS and POSAS^28^ (Table 4).

### The Stony Brook Scar Evaluation Scale (SBSES)

The SBSES was proposed in 2007 by Singer et al^29^ and is a 6-item ordinal wound evaluation scale developed to measure short-term cosmetic outcome of wounds 5 to 10 days after injury up to the time of suture removal. It incorporates assessments of individual attributes with a binary response (1 or 0) for each, as well as overall appearance, to yield a score ranging from 0 (*worst*) to 5 (*best*). The SBSES has only recently been proposed for use in research, as it was designed to measure short-term rather than long-term wound outcomes.^30^ It thus has limited applicability to pathologic scar assessment (Table 5).

Scar assessment scales have been validated to demonstrate acceptable consistency and reliability, yet they all rely on categorical or ordinal data with relatively few levels.^31^ They thus have limited sensitivity, serving to detect only large differences between scars. Individual scar attributes are all scored with equal weight. Consequently, many distinct scars can fall into the same category.

Caution must be exercised with clinical application of scar assessment scales, as they are largely subjective clinical assessments and thus highly observer dependent. The patient's own view of the scar can currently be assessed and may be very influential in determining the patient's quality of life, irrespective of the actual physical characteristics of the scar. The question remains: are any of the existent scar assessment scales superior to patient perception alone? After all, we have defined *pathologic scars* as those imparting functional impairment or nonfunctional impairment that drives a patient *to seek treatment*. Patient self-assessment of scar characterization (ie, length, width, color) has been compared with clinician evaluation without the finding of significant discrepancy.^32^ That is, follow-up visits to obtain scar data offer no benefit beyond that obtained from patient self-reported measurement and scar evaluation for the purposes of data collection for outcome measurements.

Clinical scar assessment lacks standardized methodology and a systematic approach, and thus studies continue to lack consensus regarding the most appropriate and applicable evaluation instrument. Refinement of scar assessment methods will serve to facilitate our treatment and perhaps prevention of scar formation. Factors considered in evaluation have included scar height or thickness, pliability, surface area, texture, pigmentation, and vascularity.^33^ In patient assessment, a scar should be defined in terms of its precipitating event, age, behavior since its onset, and associated symptoms (i.e, pruritus, pain). Table 1 compares and contrasts the existent scar scales and delineates some key advantages and deficiencies of each.

Future studies should be directed toward the development of a novel scar assessment scale that is applicable to hypertrophic scars causing severe disfigurement. According to arguments made in critical analysis of existent scar assessment scales, the new scale should include a patient-based component, be validated in both linear (postoperative) and burn scar populations, include psychological input, and employ clearly defined terminology and methodology for analysis.^34^

## CONCLUSION AND FUTURE DIRECTIONS

Studies are lacking that critically compare subjective scar assessment tools and objective measurement instruments with emerging devices. Elucidation of the pros and cons of each modality would greatly facilitate clinicians' usage. In addition, most current studies, classification schemes, and methods of scar evaluation have focused on burn scars. Pathologic scarring seems to have a higher prevalence after burns than after surgical procedures or other trauma. Nonetheless, few studies to date have described and analyzed the prevalence of postburn and postoperative pathologic scarring.^35^ Regarding treatment strategies, the paucity of objective and universal methods for assessing scar response to treatment has hindered progress. In addition, the molecular basis of the relationship between depth of injury and scar formation remains poorly understood. Selecting the appropriate treatment modality best suited to the type of scar assessed thus continues to pose a great challenge.

## Figures and Tables

**Table 1:** Comparison of scar assessment scales*,†

Scale	Scoring system	Attributes analyzed	Deficiencies	Advantages
**Vancouver Scar Scale**	0 to 13	Vascularity, height/thickness, pliability, and pigmentation	Lacks patient perception Pigmentation subscale less applicable to large, heterogeneous scars Operator-dependent errors Excludes pain and pruritis	Used widely in literature for outcome measure in burn studies
**Visual Analog Scale with scar ranking**	0 to 100 “excellent” to “poor”	Vascularity, pigmentation, acceptability, observer comfort *plus* contour and summing the individual scores	Photo-based scale does not include patient assessment	Simpler than VSS Assessments of intra- and interrater reliability easier to conduct
**Patient and Observer Scar Assessment Scale**	5 to 50	VSS *plus* surface area; patient assessments of pain, itching, color, stiffness, thickness, relief	Items represented may not adequately express patient's perceptions and concerns	Focuses on scar severity from clinician's and patient's points of view
**Manchester Scar Scale**	5 (*best*) to 18 (*worse*)	VAS *plus* scar color, skin texture, relationship to surrounding skin, texture, margins, size, multiplicity	Arbitrary assessment and weighting of items	Applicable to a wider range of scars Uses descriptors related to clinical significance instead of physical measurement alone
**The Stony Brook Scar Evaluation Scale**	0 (*worst*) to 5 (*best*)	VAS *plus* width, height, color, presence of suture/staple marks	Photo-based scale does not include patient assessment Not designed for long-term scar assessment	Specifically developed to assess short-term appearance of repaired lacerations

*VAS indicates Visual Analog Scale; VSS, Vancouver Scar Scale.

†None of the scar scales measure the following:

1. The amount of total body surface area that is scarred.

2. The functional disability caused by scar.

3. The effects of pain and pruritus in terms of activities of daily living.

**Table 2:** The Vancouver Scar Scale

	Scar characteristic	Score
Vascularity	Normal	0
	Pink	1
	Red	2
	Purple	3
Pigmentation	Normal	0
	Hypopigmentation	1
	Hyperpigmentation	2
Pliability	Normal	0
	Supple	1
	Yielding	2
	Firm	3
	Ropes	4
	Contracture	5
Height	Flat	0
	<2 mm	1
	2-5 mm	2
	>5 mm	3
	**Total score**	**13**

**Table 3:** Patient and Observer Scar Assessment Scale

	**Observer component**										
Normal skin	1	2	3	4	5	6	7	8	9	10	Worst scar imaginable
Vascularization											
Pigmentation											___Hypo
											___Mix
											___Hyper
Thickness											
Relief											
Pliability											
	Patient component										
No, no complaints	1	2	3	4	5	6	7	8	9	10	Yes, worst imaginable
Is the scarpainful?											
Is the scaritching?											
No, as normal skin	1	2	3	4	5	6	7	8	9	10	Yes, very different
Is the color of the scar different											
Is the scarmore stiff											
Is the thickness of the scar different?											
Is the scar irregular?											

**Table 4:** Manchester Scar Scale

	Visual Analog Scale	
Excellent	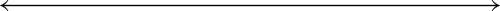	Poor
Color	Perfect	1
	Slight mismatch	2
	Obvious mismatch	3
	Gross mismatch	4
Matte vs shiny	Matte	1
	Shiny	2
Contour	Flush with surrounding skin	1
	Slightly proud/Indented	2
	Hypertrophic	3
	Keloid	4
Distortion	None	1
	Mild	2
	Moderate	3
	Severe	4
Texture	Normal	1
	Just palpable	2
	Firm	3
	Hard	4

**Table 5:** The Stony Brook Scar Evaluation Scale

	Scar category	Points
Width	>2 mm	0
	≤ 2 mm	1
Height	Elevated/depressed in relation to surrounding skin	0
	Flat	1
Color	Darker than surrounding skin	0
	Same color or lighter than surrounding skin	1
Hatch marks/Suture marks	Present	0
	Absent	1
Overall appearance	Poor	0
	Good	1
